# Development of a mammalian cell-based ZZ display system for IgG quantification

**DOI:** 10.1186/s12896-023-00798-2

**Published:** 2023-07-28

**Authors:** Lingzhi Bao, Aizhen Yang, Ziqing Liu, Jie Ma, Jiajie Pan, Yi Zhu, Ying Tang, Pu Dong, Guoping Zhao, Shaopeng Chen

**Affiliations:** 1 School of Public Health, Wannan Medical College, Wuhu, 241002 China; 2 Hefei Institutes of Physical Science, Chinese Academy of Sciences, Hefei, 230031 China

**Keywords:** Immunoglobulin G, ZZ peptide, Cell-based surface display, Quantification, Cell sorting

## Abstract

**Background:**

Biological laboratories and companies involved in antibody development need convenient and versatile methods to detect highly active antibodies.

**Methods:**

To develop a mammalian cell-based ZZ display system for antibody quantification, the eukaryotic ZZ-displayed plasmid was constructed and transfected into CHO cells. After screening by flow cytometric sorting, the stable ZZ display cells were incubated with reference IgG and samples with unknown IgG content for 40 min at 4℃, the relative fluorescence intensity of cells was analyzed and the concentration of IgG was calculated.

**Results:**

By investigating the effects of different display-associated genetic elements, a eukaryotic ZZ-displaying plasmid with the highest display efficiency were constructed. After transfection and screening, almost 100% of the cells were able to display the ZZ peptide (designated CHO-ZZ cells). These stable CHO-ZZ cells were able to capture a variety of IgG, including human, rabbit, donkey and even mouse and goat. CHO-ZZ cells could be used to quantify human IgG in the range of approximately 12.5–1000 ng/mL, and to identify high-yielding engineered monoclonal cell lines.

**Conclusions:**

We have established a highly efficient CHO-ZZ display system in this study, which enables the quantification of IgG from various species under physiological conditions. This system offers the advantage of eliminating the need for antibody purification and will contribute to antibody development.

**Supplementary Information:**

The online version contains supplementary material available at 10.1186/s12896-023-00798-2.

## Background

As ideal therapeutic and diagnostic molecules, antibodies have become the best-selling drugs in the pharmaceutical market. In recent years, the global antibody drug market has been growing at a rate of over 10% [1]. Meanwhile, major advances in the field of tumor immunotherapy vastly accelerated the expansion of antibody drugs [2]. In antibody drugs development, it is crucial to screen and identify stable and high-yielding cell lines to produce highly active antibodies [3]. Various methods have been developed to identify and characterize the performance of IgG-secreting cells, including turbidimetric assay, enzyme-linked immunosorbent assay (ELISA), mass spectrometry, high-performance liquid chromatography and surface plasmon resonance [4–7]. The quantification of antibodies using these methods necessitates antibody screening and purification, which incurs significant time and cost expenditures. Additionally, these assays are usually performed under non-physiological conditions (hostile and variable buffer environment), which may not only affect the activity of antibody, but also fail to discriminate between functional and non-functional antibodies. In the present study, to screen and identify monoclonal cells that secrete highly potent IgG, we intended to develop a novel method for IgG quantification by displaying the ZZ domain on mammalian cell surface.

The Z domain is a synthetic artificial analogue of the B domain of staphylococcal protein A [8], and ZZ are two tandem Z domains [9]. The Z peptide binds with high affinity (Kd = 10 nM) to the constant region (Fc) of the human IgG’ heavy chain and does not interfere with the antibody-antigen interaction [10]. Therefore, ZZ has been successfully applied in protein production, immunofluorescence staining and immunodetection etc [11–13]. Compared to protein A or protein G, ZZ is less time-consuming and more cost-effective. ZZ-modified beads provide a rapid method for IgG purification from serum or culture supernatants [11, 14]. Furthermore, immobilization of purified ZZ proteins on the microtiter plate or beads is an excellent immunosorbent for ELISA [11, 15, 16]. Expression and display of the Z/ZZ peptide on the surface of E. coli and yeast can eliminate the hassle of Z/ZZ purification and make the assay easier [17, 18]. However, the cell wall of these microbes is a rigid structure that constitutes a physical barrier to large molecules transit and limits the release and display of proteins [19], which may result in lower display efficiency of ZZ comparing to mammalian cells. Various of genetic elements related to ZZ display have been used to improve the display efficiency of ZZ [20]. Due to cell wall barriers and limited surface area, efficient display of Z/ZZ on microbial surfaces remains a challenge. In addition, the risk of microbial contamination weakens the application of yeast display of ZZ in the cell laboratories and biofactories.

The objective of this study was to establish a ZZ display system on the surface of mammalian cells for the quantification of highly active IgG under physiological conditions, without requiring antibody purification. We constructed a eukaryotic ZZ-displaying plasmid and transfected it into CHO cells. After flow cytometric sorting, we obtained the stable cells with high levels of ZZ display on the surface (CHO-ZZ cells). We then confirmed that these CHO-ZZ cells could be applied to the quantification of IgG and identification of high-yielding IgG expression cell lines. Due to its convenience and safety, the efficient ZZ display system has a broad application prospect in antibody development laboratories and manufacturing facilities.

## Materials and methods

### Cell culture

CHO cells (a kind gift provided by Prof. Tom K. Hei, Columbia University, USA) were cultured in DMEM/F12 medium (Gibco, USA) containing 10% fetal bovine serum (FBS, Bio-west, USA), 100 U/mL penicillin and 100 µg/mL streptomycin (Beyotime, China), at 37 °C in a 5% CO_2_ incubator.

### Vector construction

To capture antibodies on the cell surface, a series of plasmids were constructed as described below. Human elongation factor-1 alpha (EF-1 alpha) is a constitutive promoter of human origin that can be used to drive ectopic gene expression in various in vitro and in vivo contexts. Because the EF1α promoter can maintain stable and high levels of transgene expression in long-term culture compared to the CMV promoter [21, 22], we first replaced the CMV promoter of pcDNA3.1/Hygro(+) with the EF1α promoter. The synthetic EF1α promoter with *Spe*I at the 3’-end was inserted into *Mul*I-*Not*I digested pcDNA3.1/Hygro(+) to yield pEF1α. The ZZ expression plasmid, pEF1α-ZZ, was constructed by introducing a synthetic DNA containing the Ig κ chain leader and the ZZ fragment into a *Spe*I-*Not*I digested pEF1α vector. To display ZZ on cell surface, human platelet-derived growth factor receptor (PDGFR) from pDisplay or synthetic murine B7-1 was amplified with the primers (P1/P2 for PDGFR; P3/P4 for B7.1), and cloned into *Not*I digested pEF1α-ZZ to yield pEF1α-ZZ-PDGFR and pEF1α-ZZ-B7, respectively. The pET28a(+)-GFP-hTNF for GFP-hTNFα purification and phAbs1-3f8/3 for secreting full-length anti-TNFα antibody were generated in our previous study [23]. The pET28a(+)-MC-hTNF was generated by replacing GFP with mCherry at the *Nhe*I and *BamH*I sites with the primers of P5/P6 (Table 1). Plasmids were purified by Plasmid DNA Extraction Kits (Monad, China), and quantified by NanoPhotometer N50 (Implen GmbH, Germany).

**Table 1:** Primer lists in vector construction

Primers	Vectors
P1: 5’-ACTTAT**GCGGCCGC**TGTCGACACCACGCGTAATGCTGTGGGCCA GGACACGCAGGAGGTCAT-3’	pEF1α-ZZ-PDGFR
P2: 5’-AACTTC**GCGGCCGC**CTAACGTGGCTTCTTCTGCCAAAG-3’	
P3: 5’-ACATCT**GCGGCCGC**TGGCTCCGAACAAAAACTCATCTCAGAAG-3’	pEF1α-ZZ-B7
P4: 5’-AACTT**CGCGGCCGC**TAAAGGAAGACGGTCTGTTCAG-3’	
P5: 5’-ATTGCG**GCTAGC**ATGGTGAGCAAGGGCGAG-3’	pET28α(+)-MC-hTNF
P6: 5’-ATTCGC**GGATCC**CTTGTACAGCTCGTCCA-3’	

Notes: The restriction endonuclease sites are highlighted in bold

### Transfection and screening for stable cells

Transfection with polyethylenimine (PEI, Yeasen Biotechnology, China) was performed as described previously with some modifications [24]. Exponentially growing CHO cells (4 × 10^5^) were inoculated into 12-well plates and cultured for 24 h. The cells were then transfected with 2 µg of DNA and 7 µL of PEI (1 mg/mL) in Opti-MEM media. Four hours later, the transfection medium was replaced with fresh culture media. For transient transfection, the cells were passaged and cultured in complete media containing 50 µg/mL hygromycin B (Yeasen Biotechnology, China) for 48 h, then harvested and analyzed by flow cytometry. To obtain stable transfectants secreting full-length antibodies, the cells transfected with the phAbs1-3f8/3 plasmids were plated into a 100 mm dish containing 10 mL DMEM/F12 growth media with 10% FBS, penicillin, streptomycin and 700 µg/mL hygromycin B and cultured for 7 days. Colonies were picked and transferred into a 96-well plate. The media from each well were collected and centrifuged at 10,000 g for 10 min at 4 °C. The supernatants were stored at -80 °C until use.

### Cell sorting and analysis

Flow cytometers, FACSAriaIII (BD biosciences, USA) and Cytoflex S (Beckman, USA), were used for fluorescence-activated cell sorting (FACS) and analysis.

To obtain the cells with stable and high-level display of ZZ on the cell surface (CHO-ZZ), CHO cells in a 6-well plate (8 × 10^5^ cells/well) were transfected with pEF1α-ZZ-B7. Twenty-four hours later, the transfected cells were passaged on 100 mm dishes with 10 mL DMEM/F12 growth media containing 600 µg/mL hygromycin B. At 70–80% confluence, the cells were harvested and washed with cold PBS, followed by incubation with FITC-conjugated human IgG (Bersee, China, 1:400 in Optimal-MEM medium (Invitrogen, USA)) for 40 min at 4 °C. After washing with cold PBS, the cells were resuspended in cold Optimal-MEM media. 1% of living cells with high fluorescence were sorted in the purity mode, using a BD FACSAriaIII. The sorted cells were incubated in complete culture media supplemented with 50 µg/mL hygromycin B and prepared for the next round of sorting.

**Table 2:** The ability of CHO-ZZ cells to bind with fluorescein-conjugated IgG

Species	antibody	Product NO.	manufacturer
human	Human IgG-FITC	BFR550	Bersee
Rabbit	Rb pAb to 6× His tag (FITC)	Ab1206-250	Abcam
Mouse	Monoclonal ANTI-FLAG M2-FITC	F4049	Sigma
Goat	Anti-Rabbit IgG (H + L)-FITC	ZF-0311	ZSGB-BIO
Goat	Anti-mouse IgG (H + L)-FITC	ZF-0312	ZSGB-BIO
Rabbit	Rb pAb to HA tag (PE)	Ab72564	Abcam
Mouse	c-myc (9E10) PE	Sc-40 PE	Santa Cruz Biotechnology
Donkey	Donkey Anti-Rabbit IgG (H + L)-Cy3	711-165-152	Jackson ImmunoResearch Laboratories
Goat	Alexa Fluor 647 Goat anti-Mouse IgG (H + L)	A21235	Invitrogen

To analyze the performance of ZZ display, CHO-ZZ cells (2 × 10^5^ cells/sample) were labeled with a series of fluorescein-conjugated antibodies from different species (Table 2) for 40 min at 4 °C. The cells were then resuspended in the cold PBS, and the percentage of positive cells was determined using Cytoflex S (Beckman, USA).

### Immunofluorescence

The CHO-ZZ cells were seeded onto sterile cover slips. After 48 h of culture, cells were washed with PBS and fixed with 4% formaldehyde for 30 min. The fixed cells were then incubated with FITC-conjugated human IgG (1:200) in TNBS (PBS supplemented with 1% FBS and 0.1% Triton X-100) for 40 min at 4 °C. After washing with PBS, the cells were coverslipped with anti-fading agent, and the slides were imaged using a fluorescence microscope (Leica DM4B, Germany).

### Quantitation of IgG antibody with CHO-ZZ cells

Exponentially growing CHO-ZZ cells (5 × 10^5^ cells/dish) were seeded in 10 mm dishes for 2 days. Then the cells were trypsinized and collected. After washing with PBS, the cells (2 × 10^5^ cells/sample) were incubated with pre-diluted human IgG-FITC (hIgG-FITC) or target samples in 0.4 mL of FPBS (PBS containing 1% FBS) for 40 min at 4 °C. After washing and resuspension in cold PBS, cells were analyzed by flow cytometry and the mean fluorescent intensity was quantified. The concentration of hIgG-FITC was detected by ELISA assay and used as the human IgG standard. The total amount of human IgG antibody in the media was quantified by comparison with a reference standard curve (2-fold dilution series with a starting concentration of 1 µg/mL).

### Identification of high-yield stable clones

CHO-ZZ cells grown in a monolayer for 2 days were collected and aliquoted into 1.5 mL EP tubes with 3 × 10^5^ cells each. After washing with PBS, the cells were resuspended in 200 µL of basal culture media, and an equal volume of medium derived from IgG-secreting clones (for example: anti-TNFα-3f8 IgG) was added. Each sample was incubated at 4 °C for 40 min, followed by cold PBS washing. Then, the cells were incubated with GFP-TNFα or mCherry-TNFα (1:500 in FPBS) at 4 °C for 40 min. After washing and resuspension in cold PBS, the percentage of positive cells was analyzed using Cytoflex S.

### ELISA assay

The concentration of human IgG was determined using human IgG ELISA kit (Multi Sciences, China) according to the manufacturer’s instructions. The human IgG was then used as the standard for the quantitative detection of human IgG.

### Purification of GFP-TNFα and mCherry-TNFα

The recombinant proteins were expressed using E. coli BL21 (DE3) with pET28a(+)-GFP-hTNF and pET28a(+)-MC-hTNF. After induction with isopropylthiogalactoside (0.5 mM) at 25 °C overnight, the cells were sonicated and centrifuged at 16,000 g for 30 min. The supernatant was loaded into Ni-NTA gravity column containing 1 mL bead volume (Smart-Lifesciences, China). Recombinant proteins were eluted in lysis buffer containing 80 mM imidazole in binding buffer (500 mM NaCl, 20 mM Tris-Cl, 15% glycerol, pH 7.5). The collected proteins were dialyzed against PBS, concentrated with an Amicon Ultra 10 kDa filter (Merk Millpore, Germany), and subjected to SDS-PAGE. The concentration of the purified protein was determined using BCA protein assay kit (Beyotime, China) according to the manufacturer’s instructions.

### Data analysis

**Fig. 1:**
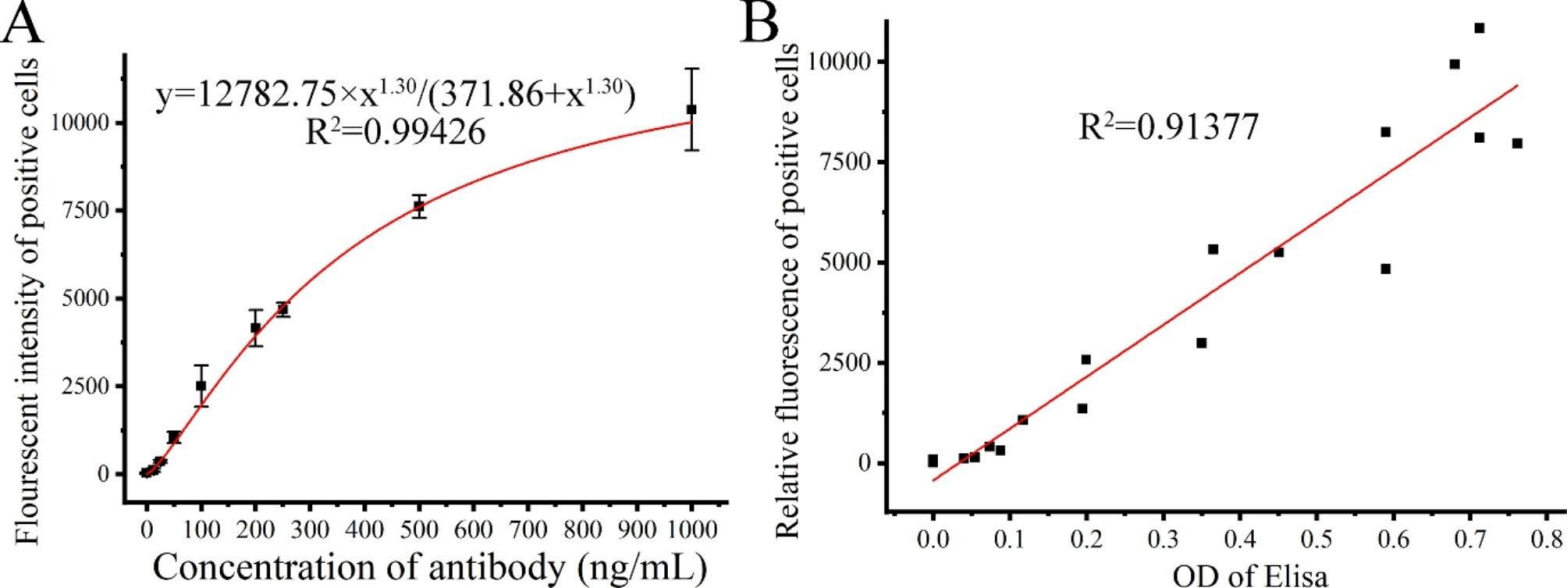
A plotted standard curve for IgG quantification with CHO-ZZ- cells and its reliability. (**A**) A non-linear regression plot of the standard curve for IgG quantification. CHO-ZZ cells were incubated with the FITC-conjugated hIgG in the range of 12.5–1000 ng/mL for 40 min at 4℃, and analyzed by flow cytometry. The data were well fitted with three-parameter Hill functions. (**B**) Linear relationship between IgG binding activity and OD of ELISA. The relative fluorescence intensity of positive cells and the concentration of IgG showed a strong positive correlation. Coefficient of determination (R^2^) is shown

The resulting data (Fig. 1A) were fitted to a Hill function model to establish the relationship between the fluorescence intensity of the positive cells and the concentration of IgG [25]. The equation used for curve fitting is:

$$y={V}_{max}\times {x}^{n}/\left({k}^{n}+{x}^{n}\right)$$

where y is the fluorescence intensity of positive cells, which was calculated by multiplying the percentage of positive cells by their average fluorescence intensity, x is the concentration of the IgG, Vmax is the maximum fluorescence level responding to IgG, k and n are the Hill constant and coefficient, respectively.

The results of the quantitative analysis are presented as means with standard deviations (SD) of three separate assays. Significance levels were assessed by Student’s t-test and set at p ≤ 0.05.

## Results and discussion

### Surface display of ZZ peptide on the CHO cell for IgG capture

**Fig. 2:**
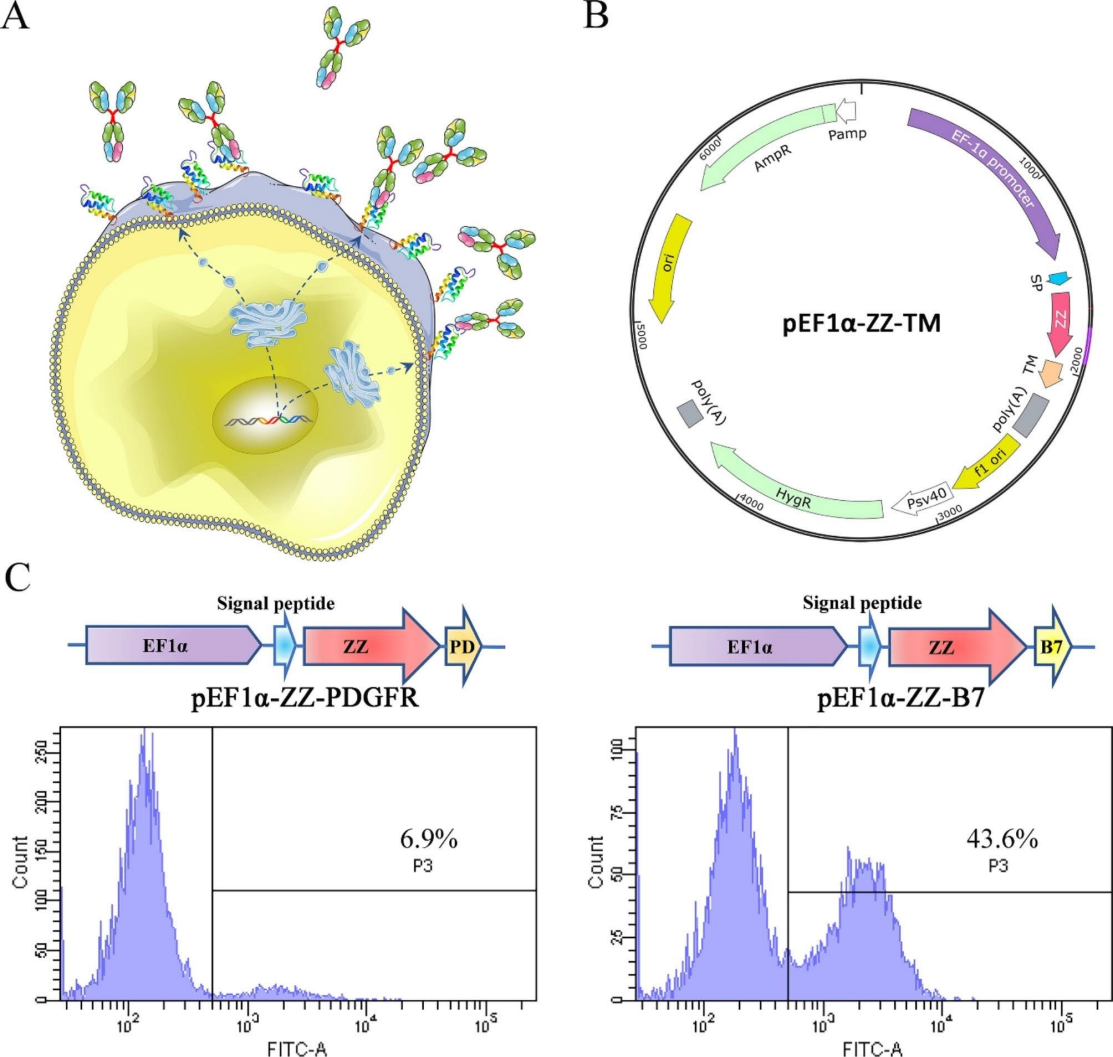
Displaying ZZ on the surface of CHO cell to capture IgG antibodies. (**A**) Schematic illustrations of capturing IgG with CHO cells displaying ZZ peptides. (**B**) Scheme of the ZZ display vectors. SP, signal peptide; ZZ, two tandem Z domains of protein A; TM, transmembrane domain. (**C**) The scaffold design and display efficiency of pEF1α-ZZ-PDGFR and pEF1α-ZZ-B7. CHO cells were transfected with pEF1α-ZZ-PDGFR and pEF1α-ZZ-B7, respectively. One day later, the cells were collected, labeled with FITC-hIgG, and then analyzed by flow cytometry

The capture of IgG antibodies by displaying ZZ peptide in the periplasm of E. coli and on yeast surface has already been documented in the literature [18, 20, 26]. Here, to quantitatively identify functional IgG products under physiological conditions, we displayed the ZZ peptide on the surface of CHO cells. We designed and constructed a series of eukaryotic plasmids with different display-associated genetic elements, including CMV and EF1α promoters, signal peptides (SP), ZZ domain, and transmembrane region (TM). After transfection with ZZ-containing plasmids, the ZZ peptides were displayed on the surface of CHO cell. These ZZ display cells were used to capture and quantify IgG antibodies by labeling with fluorescein- or enzyme-conjugated antibodies or antigens (Fig. 2A and B). The primary consideration for quantifying IgG in this manner is the efficiency of the ZZ display. The transgene expression efficiency of the plasmids constructed by the EF1α promoter and Igκ light chain signal peptide (Mus musculus) is better than that of the CMV promoter and serum albumin preproprotein (Homo sapiens) [22, 27]. Considering that protein expression driven by the EF1α promoter was maintained longer than that driven by the CMV promoter [28], the EF1α promoter and Igκ light chain signal peptide were chosen to display the ZZ peptide. We also compared the efficiency of ZZ display on CHO cell surface with different transmembrane domains (TM). Human PDGFR and murine B7-1 TM were chosen to generate pEF1α-ZZ-PDGFR and pEF1α-ZZ-B7 plasmids, respectively. Two days after transfection, cells were labeled with hIgG-FITC. As shown in Fig. 2C, the percentage of ZZ-positive cells in pEF1α-ZZ-B7 transfected cells is higher than pEF1α-ZZ-PDGFR transfected cells (43.6% vs. 6.9%). This indicates that B7-1 TM is more conducive to ZZ display on the CHO cell surface than PDGFR TM, which is consistent with the reported literature [29, 30]. Consequently, pEF1α-ZZ-B7 was used to display ZZ on the cell surface for the following experiments.

### Screening the cells with efficiently displayed ZZ

**Fig. 3:**
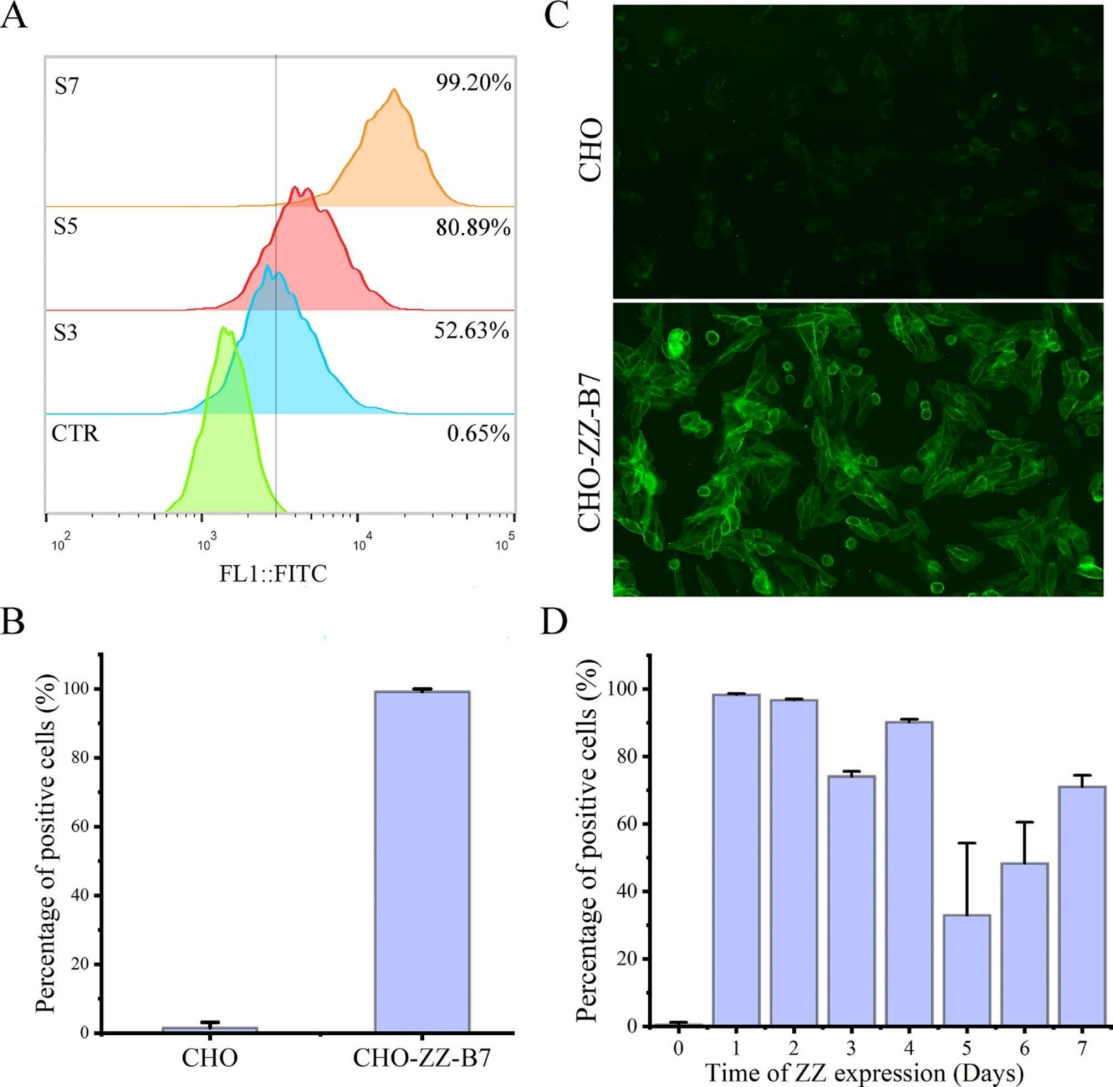
Cell sorting for stable CHO-ZZ cells. (**A**) Levels of ZZ display on the surface of the cells from the third-, fifth-, and seventh-round of sorting. (**B**) Percentage of ZZ-positive cells in the seventh round. (**C**) Identification of ZZ display by immunofluorescence imaging. (**D**) Time-course ZZ display of CHO-ZZ cells. The data was pooled from three independent experiments. CHO-ZZ cells were harvested, labelled with FITC-hIgG and then analysed by flow cytometry. For imaging, CHO-ZZ cells were seeded on the coverslips for two days. The cells were then fixed with 4% formaldehyde, labelled with FITC-hIgG and imaged by microscopy

The efficiency of ZZ display was low and unstable in transient transfection experiments, which did not meet the requirements of IgG quantification. To address this issue, we attempted to screen the transformants using flow cytometric sorting. CHO cells were transfected with pEF1α-ZZ-B7 and stained with hIgG-FITC. The top 1% of cells with the highest FITC fluorescence were sorted in the purity mode. As the sorting progressed, more ZZ-positive cells with higher fluorescence were obtained (Fig. 3A). After seven rounds of sorting, the percentage of ZZ-positive cells reached to almost 100% (Fig. 3B). Furthermore, the cells were quite stable, and the display of ZZ was maintained at a high level during the long-term cultures (approximately 3 months, Fig. S1). To confirm the display of ZZ on the CHO cell surface, the cells were then stained with immunofluorescence and imaged using microscopy. Figure 3 C showed that ZZ peptides were displayed on the surface of the cells. After the cells were seeded, ZZ display rapidly reached to a peak level and maintained for 2 days. Thereafter, ZZ display fluctuated, with only 30% of positive cells on the fifth day. However, the loss of ZZ display can be recovered by replacing the exhausted medium with fresh medium, suggesting that CHO-ZZ cells were best for use within 2 days after passage (Fig. 3D). These results indicate that CHO-ZZ cells we obtained can efficiently and stably display ZZ peptides.

### Capability of CHO-ZZ cells to capture different species-derived antibodies

**Fig. 4:**
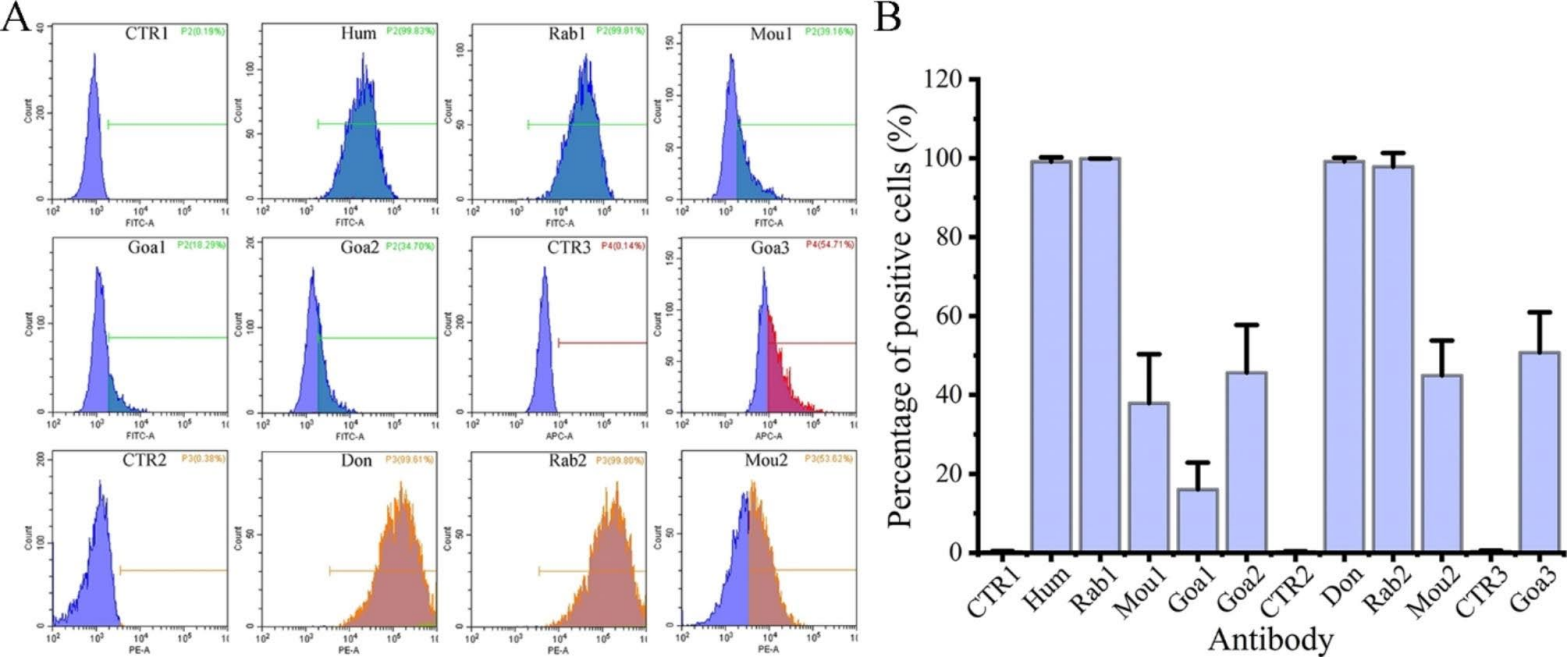
The capturing capability of CHO-ZZ cell to the different species-derived IgG. (A) The histograms of CHO-ZZ cells with IgG capture. (B) The percentage of positive CHO-ZZ cells labeled with fluorescein-conjugated IgG. CHO-ZZ and CHO cells were incubated with an excess of fluorescein-conjugated IgG listed in Table 2 for 40 min at 4℃, washed by PBS, and analyzed by flow cytometry. Hum: human, Rab: rabbit, Goa: goat, Don: donkey, and Mou: Mouse. The data are pooled from three independent experiments, CTR1: CHO cells labeled with FITC-conjugated antibody (a mixture containing human, rabbit, and mouse IgG), CTR2: CHO cells labeled with PE- and cy3-conjugated antibody (a mixture containing donkey, rabbit and mouse IgG), and CTR3: CHO cells labeled with Alexa Fluor 647

The ZZ peptides interact with IgG from a variety of mammals including human and rabbit [17]. We characterized the IgG capture capability of CHO-ZZ cells by using antibodies originated from different species. After incubation for 40 min with an excess of fluorescein-conjugated IgG as listed in Table 2, the cells were analyzed by flow cytometry. As shown in Fig. 4, CHO-ZZ cells had a stronger ability to capture human, rabbit and donkey IgG, and the percentage of positive cells labeled with these antibodies reached almost 100%. It was reported that the ZZ peptide interacted with human IgG and rabbit IgG on the yeast surface, but not with goat IgG [20]. However, our isolated CHO-ZZ cells could capture the IgG derived from goat and mouse with the percentage of positive cells between 16 and 45%. These data confirm that ZZ binds to mouse and goat IgG, but only weakly due to low affinity [31]. The low density of ZZ on the surface of microbes makes it difficult to capture mouse and goat IgG on their surface. The detectable of our display system to mouse and goat IgG might due to the efficient display of high levels of ZZ on mammalian cell surface. The result indicates that CHO-ZZ cells can capture IgG in a wide range of mammals, and the capture capability of CHO-ZZ cells is related to the species origin of the IgG.

### Quantification of IgG with ZZ display cells

One of the main purposes of screening for stable CHO-ZZ cells is to quantitatively detect IgG under physiological conditions. CHO-ZZ cells were incubated with the FITC-conjugated hIgG in the range of approximately 12.5–1000 ng/mL for 40 min at 4 °C. In the previous experiments, we had determined the time-response relationship between hIgG-FITC and the CHO-ZZ cells and found that the capture of hIgG on the surface of CHO-ZZ cells reached a plateau after incubation for 30 min (data not shown). In addition, although CHO-ZZ cells could capture IgG over a wide temperature range (4–37 °C), we still performed the experiments at 4 °C to prevent the elevated fluorescence background resulted from cell uptake of the fluorescent dyes. To achieve stable binding of ZZ to IgG, the CHO-ZZ cells were incubated with IgG for 40 min at 4 °C. ZZ peptides captured IgG in a dose-dependent manner. A standard curve for IgG quantification was generated by plotting the fluorescence intensity of positive cells against the concentration of IgG standards and fitted by a non-linear regression with an R^2^ value of 0.99426 (Fig. 1A).

The ELISA is the gold standard for antibody quantification. To test the reliability of IgG quantification using CHO-ZZ cells, we further confirmed the correlation between cell fluorescence and IgG concentration by ELISA. Figure 1B showed that the relative fluorescence values correlated well with optical densities (OD) in the ELISA assay, and the correlation coefficient was 0.91377. These results suggest that CHO-ZZ cells can be used for IgG quantification efficiently.

### Identification of stable CHO clones producing high yields of antibody

**Fig. 5:**
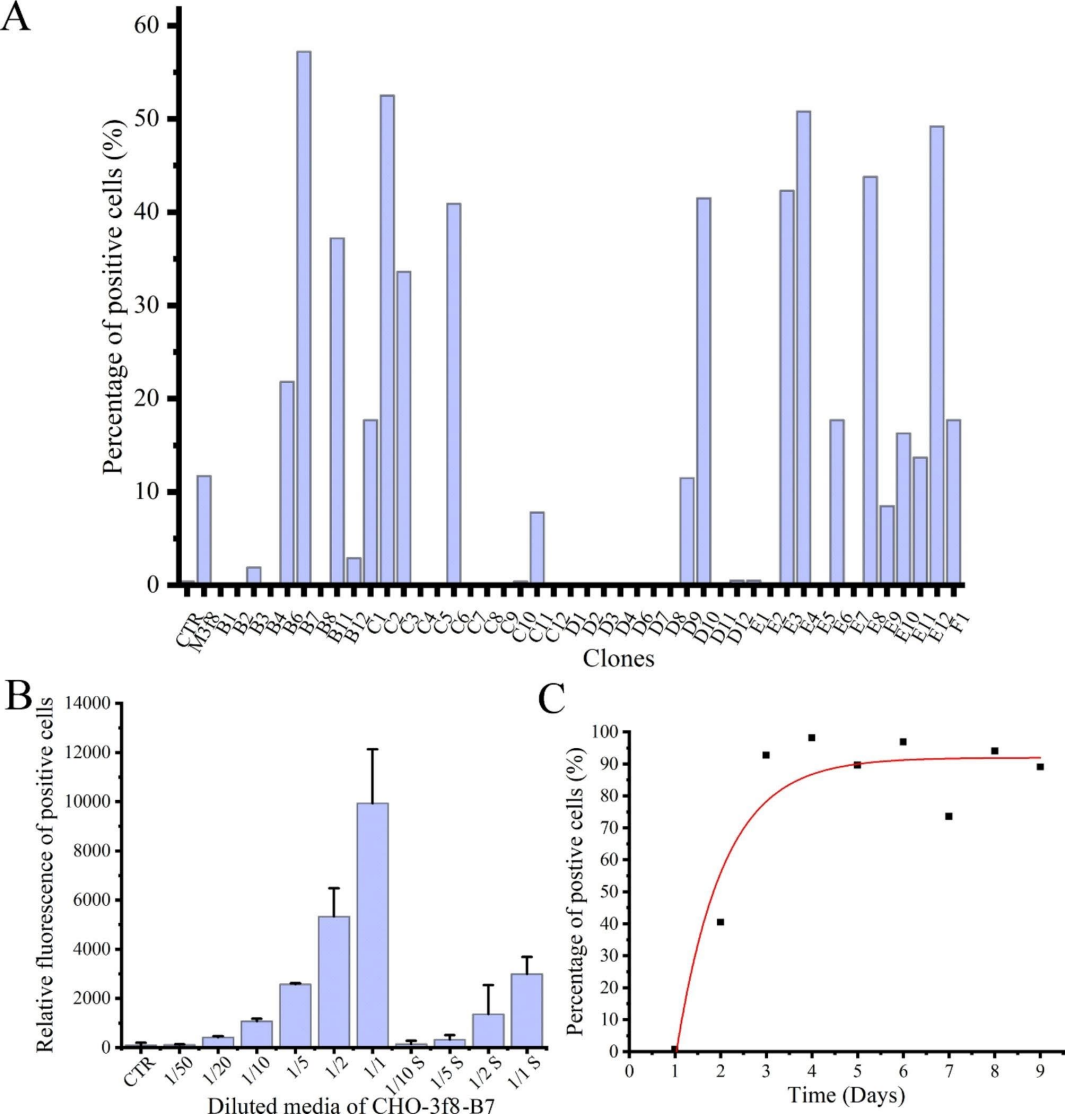
Identification of monoclonal cells for the high-efficiency production of human anti-TNFα IgG (3f8). (**A**) The positive cells labeled by the culture media from 45 clones. (**B**) The dose-dependent IgG binding activity of CHO-ZZ cells to the culture media. CHO-ZZ cells were labeled with a series of diluted media collected from the attached and suspended 3f8-B7 cells. S: media from suspension 3f8-B7 cells. (**C**) The time curve of IgG secreting by attached 3f8-B7 cells

It is well known that screening of stable, high-yielding hybridoma or engineered monoclonal cells is essential for the production of high-quality monoclonal antibodies. Here, we used ZZ display system to isolate and identify engineered monoclonal cells with high productivity of human anti-TNFα IgG. CHO cells were transfected with phAbs1-3f8/3 plasmids and selected with hygromycin B for 7 days, the media derived from IgG secreting clones were used to label CHO-ZZ cells. After staining with mCherry-TNFα, the binding capability of human anti-TNFα IgG derived from each clone to CHO- ZZ cells were determined. We examined 45 samples and found that 21 samples showed different levels of surface staining positive CHO-ZZ cells. The sample from clone 3f8-B7 had the highest binding activity (Fig. 5A). We then collected the media from 3f8-B7 adherent and suspension cells, respectively, and labeled CHO-ZZ cells with a series of diluted 3f8-B7 media. The fluorescence response presented a good correlation with dilution, and the active IgG level of 3f8-B7 adherent cells was higher than that of suspension cells (Fig. 5B). Finally, we investigated the kinetics of IgG secretion using CHO-ZZ cells. Media from 3f8-B7 adherent cells were collected every day and used to label CHO-ZZ cells. Figure 5 C showed that IgG secretion levels increased rapidly and plateaued on the third day. These results indicate that CHO-ZZ cells can be used to identify and characterize the IgG-secreting cells.

Compared to conventional ELISA assays, detection using the ZZ display system is more convenient as it eliminates the need for antibody purification in cell laboratories and biofactories. Besides, all the detection steps of the ZZ display system are performed under mild conditions (similar to the physiological environment), which helps to improve the detection accuracy for isolating monoclonal cells that secret highly active IgG. In addition, the ZZ display system can self-generate and immobilize ZZ on the cell surface, thus eliminating the tedious laborious and time-consuming procedures of ZZ purification and immobilization. However, this method is limited to the detection of active/functional antibodies due to its reliance on fluorescent antigens.

Compared to microbial display, the mammalian cell display system enhances the efficiency of ZZ display and mitigates the risk of microbial contamination in cell laboratories and biofactories. However, its primary drawback is that cultivating cells is a time-consuming and labor-intensive process. Our upcoming research will concentrate on the development of technologies for large-scale production and preservation of CHO-ZZ cells while maintaining their detection efficiency. This will help to link mammalian cell display system with high-throughput and automated techniques. With further ongoing research in the future, this system may be used in other areas such as immunoassay and immunodetection, high throughput cell sorting, etc.

## Conclusions

This work reported an efficient cell-based ZZ display system for IgG detection, including (1) the construction of ZZ display plasmids and their transfection into mammalian cells, and (2) the isolation of stable cells with high levels of ZZ display (CHO-ZZ cells) using fluorescence activated cell sorting. These ZZ display cells presented potential applications in IgG quantification and identification of high-yielding engineered monoclonal cells, which will attract more attention in cell laboratories and biofactories.

## Electronic supplementary material

Below is the link to the electronic supplementary material.

Supplementary Material 1

Supplementary Material 2

## Data Availability

All data generated or analyzed during this study are included in this published article and Supplementary file.

## Abbreviations

IgG: immunoglobulin G
ELISA: enzyme-linked immunosorbent assay
FACS: fluorescence-activated cell sorting
PEI: polyethylenimine
SP: signal peptides
TM: transmembrane domain
OD: optical density
