# Supplementary material for: Development of a mammalian cell-based ZZ display system for IgG quantification
Source: BMC Biotechnol. 2023 Jul 28;23:24. doi: 10.1186/s12896-023-00798-2 (PMC10375748; doi:10.1186/s12896-023-00798-2)
Supplement: Supplementary file 1 — Supplementary Material 1 [file 12896_2023_798_MOESM1_ESM.docx]

## Development of a mammalian cell-based ZZ display system for IgG quantification

Lingzhi Bao^1^, Aizheng Yang^1^, Ziqing Liu^1^, Jie Ma^1^, Jiajie Pan^1^, Yi Zhu^1^, Ying Tang^1^, Pu Dong^1^, Guoping Zhao^2^, and Shaopeng Chen^1^*

^1^School of Public Health, Wannan Medical College, Wuhu 241002, China;

^2^Hefei Institutes of Physical Science, Chinese Academy of Sciences, Hefei 230031, China;

* Corrospondence: Shaopeng Chen, [20200080@wnmc.edu.cn](mailto:20200080@wnmc.edu.cn)

**Figure S1**


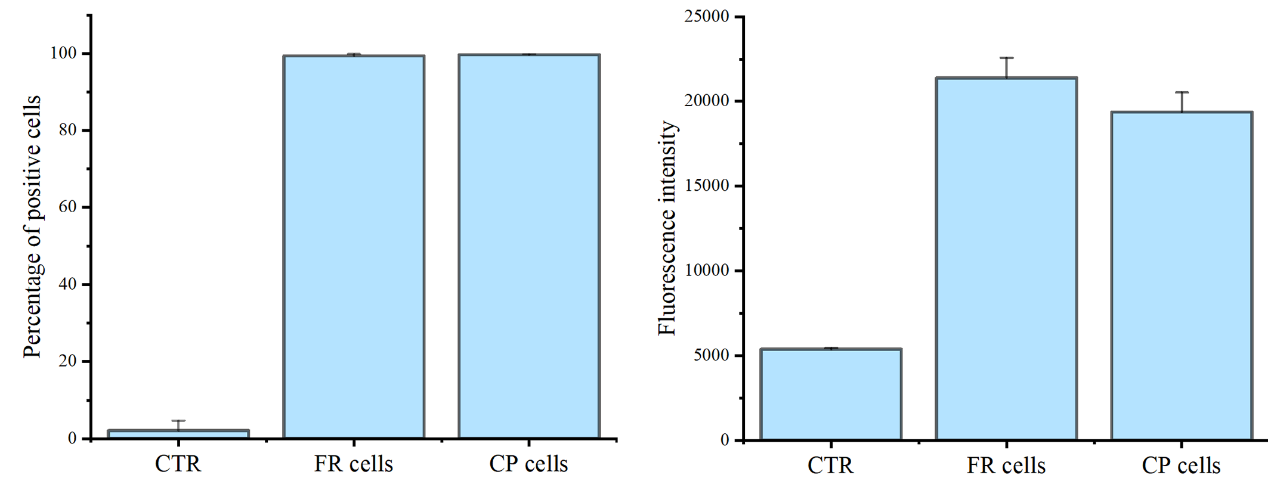


Figure S1. The effects of passage on the ZZ display. To investigate the stability of ZZ display, the freshly revived (FR) and continuously passaged (CP) CHO-ZZ cells (more than 3 months) were labelled and analyzed by flow cytometry. (A) The percentage of positive cells. (B) The relative fluorescent intensity of positive cells.
