# Supplementary material for: Development of a mammalian cell-based ZZ display system for IgG quantification
Source: BMC Biotechnol. 2023 Jul 28;23:24. doi: 10.1186/s12896-023-00798-2 (PMC10375748; doi:10.1186/s12896-023-00798-2)
Supplement: Supplementary file 2 — Supplementary Material 2 [file 12896_2023_798_MOESM2_ESM.docx]

## Development of a mammalian cell-based ZZ display system for IgG quantification

Lingzhi Bao^1^, Aizheng Yang^1^, Ziqing Liu^1^, Jie Ma^1^, Jiajie Pan^1^, Yi Zhu^1^, Ying Tang^1^, Pu Dong^1^, Guoping Zhao^2^, and Shaopeng Chen^1^*

^1^School of Public Health, Wannan Medical College, Wuhu 241002, China;

^2^Hefei Institutes of Physical Science, Chinese Academy of Sciences, Hefei 230031, China;

* Corrospondence: Shaopeng Chen, [20200080@wnmc.edu.cn](mailto:20200080@wnmc.edu.cn)

**Original data of Figure 2A**


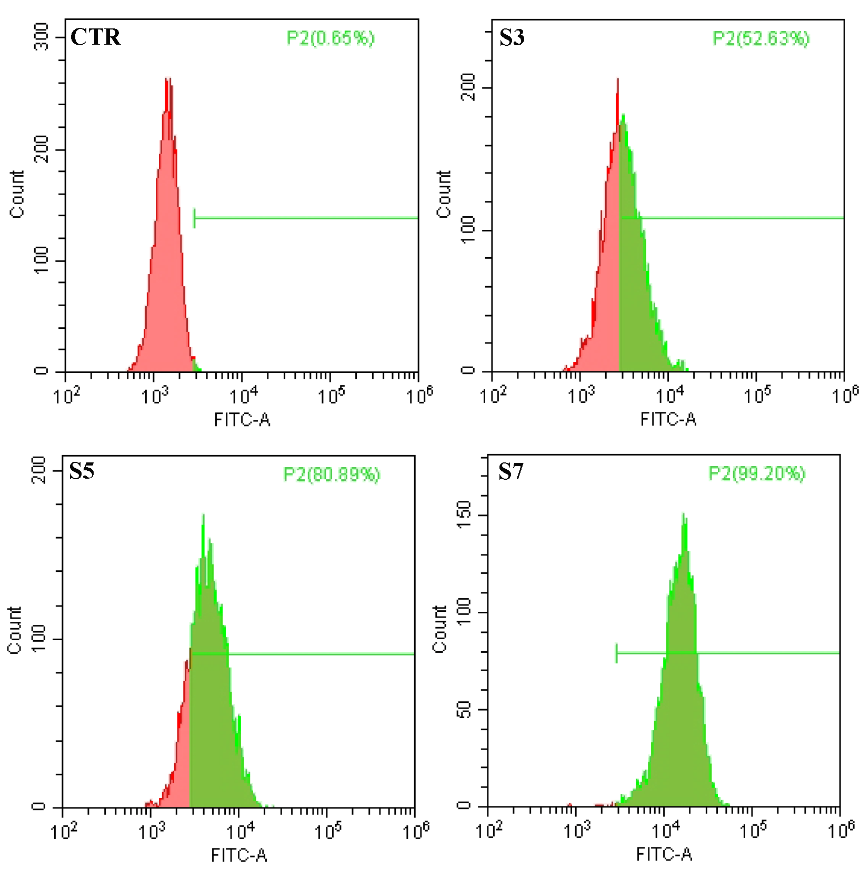


**Figure legends:** Levels of ZZ display on the surface of the cells from the third-, fifth-, and seventh-round of sorting.

**Original data of Figure 2B**

Table 1. Percentage of ZZ-positive cells in the seventh round.

| Cells | Percentage of positive cells (%), n=5 | | | | |
| --- | --- | --- | --- | --- | --- |
|  | 1 | 2 | 3 | 4 | 5 |
| CHO | 0.85 | 4.05 | 0.74 | 1.99 | 0.06 |
| CHO-ZZ | 99.2 | 99.47 | 99.7 | 97.87 | 99.74 |

**Original data of Figure 2D**

Table 2. Time-course ZZ display of CHO-ZZ cells.

| Cells | Time | Percentage of positive cells (%), n=3 | | |
| --- | --- | --- | --- | --- |
|  |  | 1 | 2 | 3 |
| CHO | 1 Day | 1.46 | 0.11 | 0.04 |
| CHO-ZZ | 1 Day | 98.69 | 97.98 | 98.22 |
|  | 2 Days | 96.92 | 96.32 | 96.79 |
|  | 3 Days | 73.97 | 72.62 | 75.68 |
|  | 4 Days | 89.69 | 89.77 | 91.1 |
|  | 5 Days | 8.3 | 45.07 | 45.5 |
|  | 6 Days | 34.16 | 55.02 | 55.71 |
|  | 7 Days | 68.59 | 73.45 | -- |

**Original data of Figure 3B**

Table 3. The percentage of positive CHO-ZZ cells labeled with fluorescein-conjugated IgG.

|  | Percentage of positive cells (%), n≥3 | | | |
| --- | --- | --- | --- | --- |
|  | 1 | 2 | 3 | 4 |
| CTR1 | 0.26 | 0.16 | 0.19 | 0.32 |
| Hum | 99.77 | 99.41 | 99.83 | 97.52 |
| Rab1 | 99.88 | 99.92 | 99.81 | -- |
| Mou1 | 54.88 | 30.59 | 39.16 | 26.93 |
| Goa1 | 9.72 | 11.38 | 18.29 | 24.63 |
| Goa2 | 59.68 | 36.34 | 34.7 | 51.74 |
| CTR2 | 0.19 | 0.19 | 0.38 | -- |
| Don | 98.08 | 99.78 | 99.61 | -- |
| Rab2 | 93.84 | 99.89 | 99.8 | -- |
| Mou2 | 35.85 | 45.23 | 53.62 | -- |
| CTR3 | 0.27 | 0.14 | 0.61 | -- |
| Goa3 | 58.38 | 54.71 | 39.14 | -- |

**Original data of Figure 4A**

Table 4. The dose response of CHO-ZZ cells.

| hIgG-FITC  (ng/mL) | 1 | | 2 | | 3 | |
| --- | --- | --- | --- | --- | --- | --- |
|  | percentage | fluorescence | percentage | fluorescence | percentage | fluorescence |
| 0 | 0.74 | 1766 | 1.79 | 1664.2 | 2.06 | 2670.2 |
| 12.5 | 3.97 | 1731 | 5.78 | 1688.1 | 6.99 | 2420.7 |
| 25 | 19.47 | 1855.4 | 17.83 | 1782.4 | 16.45 | 2478.5 |
| 50 | 54.25 | 2135 | 43.25 | 1991.9 | 41.16 | 2710.6 |
| 100 | 92.69 | 3426 | 80.67 | 2593.8 | 70.19 | 3208.6 |
| 200 | 98.12 | 4839.3 | 95.78 | 3985.7 | 89 | 4377.7 |
| 250 | 98.44 | 4986 | 98.15 | 4614.8 | 93.22 | 4942.5 |
| 500 | 99.36 | 7408.9 | 99.47 | 8022.1 | 97.87 | 7671.1 |
| 1000 | 99.71 | 9306.6 | 99.74 | 10279.7 | 99.55 | 11640.8 |

**Original data of Figure 4B**

Table 5. Linear relationship between IgG binding activity and OD of ELISA.

| OD | RFU | OD | RFU | OD | RFU |
| --- | --- | --- | --- | --- | --- |
| 0 | 91.40425 | 0.67975 | 9932.04983 | 0.712 | 8100.79356 |
| 0.04015 | 112.40545 | 0.0544 | 142.78772 | 0.5902 | 4830.00646 |
| 0.0735 | 413.27801 | 0.08765 | 319.0892 | 0.7612 | 7960.79826 |
| 0.11705 | 1067.67771 | 0.1946 | 1355.33041 | 0.4505 | 5250.34624 |
| 0.19935 | 2570.72807 | 0.3499 | 2989.2629 | 0.712 | 10838.12308 |
| 0.36535 | 5325.30438 | 0 | 14.40048 | 0.5902 | 8248.4596 |

RFU: Relative fluorescence of positive cells; OD: Optical density of Elisa.

**Original data of Figure 5A**

Table 6. The positive cells labeled by the culture media from 45 clones.

| Clone Number | Percentage of positive cells | Clone Number | Percentage of positive cells | Clone Number | Percentage of positive cells |
| --- | --- | --- | --- | --- | --- |
| CTR | 0.4 | C6 | 40.9 | D11 | 0.2 |
| M3f8 | 11.7 | C7 | 0.1 | D12 | 0.5 |
| B1 | 0.1 | C8 | 0.1 | E1 | 0.5 |
| B2 | 0.1 | C9 | 0.1 | E2 | 0.2 |
| B3 | 1.9 | C10 | 0.4 | E3 | 42.3 |
| B4 | 0.1 | C11 | 7.8 | E4 | 50.8 |
| B6 | 21.8 | C12 | 0.2 | E5 | 0.2 |
| B7 | 57.2 | D1 | 0.1 | E6 | 17.7 |
| B8 | 0.1 | D2 | 0.1 | E7 | 0.1 |
| B11 | 37.2 | D3 | 0 | E8 | 43.8 |
| B12 | 2.9 | D4 | 0 | E9 | 8.5 |
| C1 | 17.7 | D6 | 0.1 | E10 | 16.3 |
| C2 | 52.5 | D7 | 0.1 | E11 | 13.7 |
| C3 | 33.6 | D8 | 0.1 | E12 | 49.2 |
| C4 | 0.1 | D9 | 11.5 |  |  |
| C5 | 0.2 | D10 | 41.5 |  |  |

**Original data of Figure 5B**

Table 7. IgG binding activity of CHO-ZZ cells to diluted culture media.

| Dilluted media | Relative fluorescence of positive cells | |
| --- | --- | --- |
| CTR | 2168.45 | 16112.4 |
| CTRL | 9887.177 | 12593.912 |
| 1/50 | 37514.43 | 45141.173 |
| 1/20 | 99793.31 | 113742.232 |
| 1/10 | 253968.088 | 260177.526 |
| 1/5 | 613681.44 | 451379.436 |
| 1/2 | 1.14833E6 | 838080.264 |
| 1/1 | 24030.945 | 4526.6 |
| 1/10S | 45199.98 | 18617.86 |
| 1/5S | 219385.413 | 51680.67 |
| 1/2S | 347810.76 | 250041.82 |
| 1/1S | 2168.45 | 16112.4 |

**Original data of Figure 5C**

Table 8. The time curve of IgG secreting by attached 3f8-B7 cells.

|  | Time of antibody production (Days) | | | | | | | | |
| --- | --- | --- | --- | --- | --- | --- | --- | --- | --- |
|  | 1 | 2 | 3 | 4 | 5 | 6 | 7 | 8 | 9 |
| Percentage of positive cells | 0.77 | 40.47 | 92.75 | 98.13 | 89.61 | 96.9 | 73.56 | 94 | 89.05 |

**Original data of Figure S1**

Table 9. The effects of passage on the ZZ display.

| Cells | 1 | | 2 | | 3 | |
| --- | --- | --- | --- | --- | --- | --- |
|  | percentage | fluorescence | percentage | fluorescence | percentage | fluorescence |
| CTR | 5.08 | 5297.3 | 0.49 | 5417.2 | 0.58 | 5409.6 |
| FR | 99.8 | 22086 | 99.37 | 22052.6 | 98.85 | 20027.5 |
| CP | 99.8 | 20252.8 | 99.56 | 19807.2 | 99.67 | 18049.3 |
